# Negative Pressure Disinfection and Obturation of a Mandibular Premolar With Type IIIb Dens Invaginatus: Case Report and Treatment Considerations

**DOI:** 10.1155/crid/9390436

**Published:** 2025-12-11

**Authors:** Antonis Chaniotis, Anastasia Chanioti

**Affiliations:** ^1^ Dental School, Department of Endodontics, National and Kapodistrian University of Athens, Athens, Greece, uoa.gr

**Keywords:** bioceramic sealers, dens invaginatus, microscopy, vacuum disinfection, vacuum obturation

## Abstract

Dens invaginatus presents complex anatomical challenges in endodontic treatment, particularly in its severe form, Type III, where the invagination extends through the root and into the periapical tissue. The intricate morphology often complicates effective disinfection and obturation, leading to potentially higher treatment failure rates. The present case describes the noninstrumentation root canal treatment of a dens invaginatus Type IIIb in a second mandibular premolar associated with apical periodontitis. After CBCT mapping of the complex anatomy, modified negative pressure irrigation and passive ultrasonic activation were used to disinfect the complex anatomy. A CBCT‐assisted negative pressure bioceramic obturation technique was used to seal the extraordinary anatomy. The 5‐year CBCT follow‐up shows healing of the periapical lesion and the optimal distribution of the obturation material in the highly complex anatomy.

**Key Learning Points**

• Thorough clinical and CBCT radiographic examination is required to diagnose and plan the treatment of severe invaginations.
• Simple clinical techniques incorporating negative pressure and ultrasonic activation (UA) may facilitate the obturation of severe invaginations with interconnecting anatomy.

## 1. Introduction

Dens invaginatus (DI) is a rare dental anomaly that occurs during tooth development, where the enamel organ invaginates into the dental papilla before calcification occurs [1, 2]. It is among the most prevalent developmental tooth anomalies, with a prevalence ranging from 1% to 10% [1]. This malformation is most commonly seen in maxillary lateral incisors [1, 3], although it can also affect other teeth [4]. Due to the invagination, the tooth is highly susceptible to early carious lesions and pulp infections.

The endodontic treatment of DI presents unique challenges. The complexity of the internal anatomy often leads to early pulp involvement, which can result in periapical pathology. Depending on the extent of the invagination, root canal treatment can be highly complex, necessitating the use of advanced techniques such as microscopy or cone beam computed tomography (CBCT) for accurate visualization of the root canal anatomy [5], supplemented by innovative disinfection and obturation modifications.

According to the depth of the invagination, DI is divided into three types. Types I and II are confined in the crown and root of the teeth without any communication with the periodontal ligament. On the other hand, in Type III, the invagination passes through the root and communicates with the periodontal ligament, laterally in Type IIIa and apically in Type IIIb [6]. Among all types of DI, Type IIIb represents the most complex variation, where the invagination extends through the center of the root, creating an open communication with the periapical tissue [6, 7]. This severe form often leads to early pulpal necrosis and periapical/peri‐invagination pathology, making management particularly challenging. The intricate anatomy of these cases requires a comprehensive approach to disinfection, shaping, and obturation, as traditional root canal methods may not adequately address the complexity of the canal system [8].

This case report introduces the concept of the modified negative pressure bioceramic obturation technique for the safe and predictable sealing of interconnecting anatomical root canal spaces following noninstrumentation disinfection strategies applied in a case of Type IIIb DI.

## 2. Case Report

**Figure 1:**
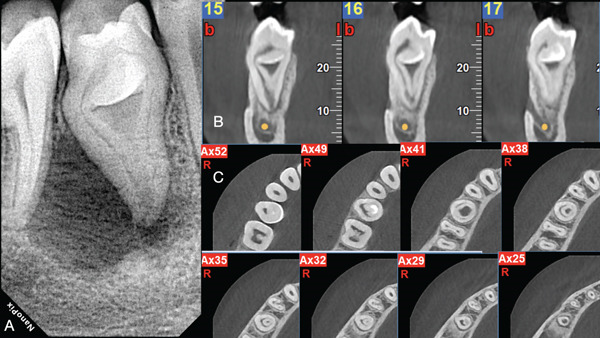
(A) Preoperative periapical radiograph revealing the Type IIIb dens invagination. (B) Sagittal CBCT Slices 15, 16, and 17 of the dens in dente Type IIIb revealing the buccolingual topography of intracanal communications. (C) Axial slices revealing the initial entry access point (Ax52), the strategically extended access (Ax49), the access point to the invagination and the c‐shaped canal spaces in the periphery of the invagination (Ax41), the c‐shaped root canal surrounding the invagination (Ax38, 35, 32), and the apical topography of the interconnecting periphery located canal with the central invagination space (Ax29,25).

A 16‐year‐old Caucasian male patient was referred for the evaluation and possible treatment of his right second mandibular premolar, suffering from pulp necrosis with an acute apical abscess. The medical history was noncontributory. In the dental history, the young patient reported that 3 years ago, he had been subjected to a presurgical CBCT examination to extract his lower third molars. The CBCT examination revealed a DI second mandibular premolar (Figure 1B,C).

**Figure 2:**
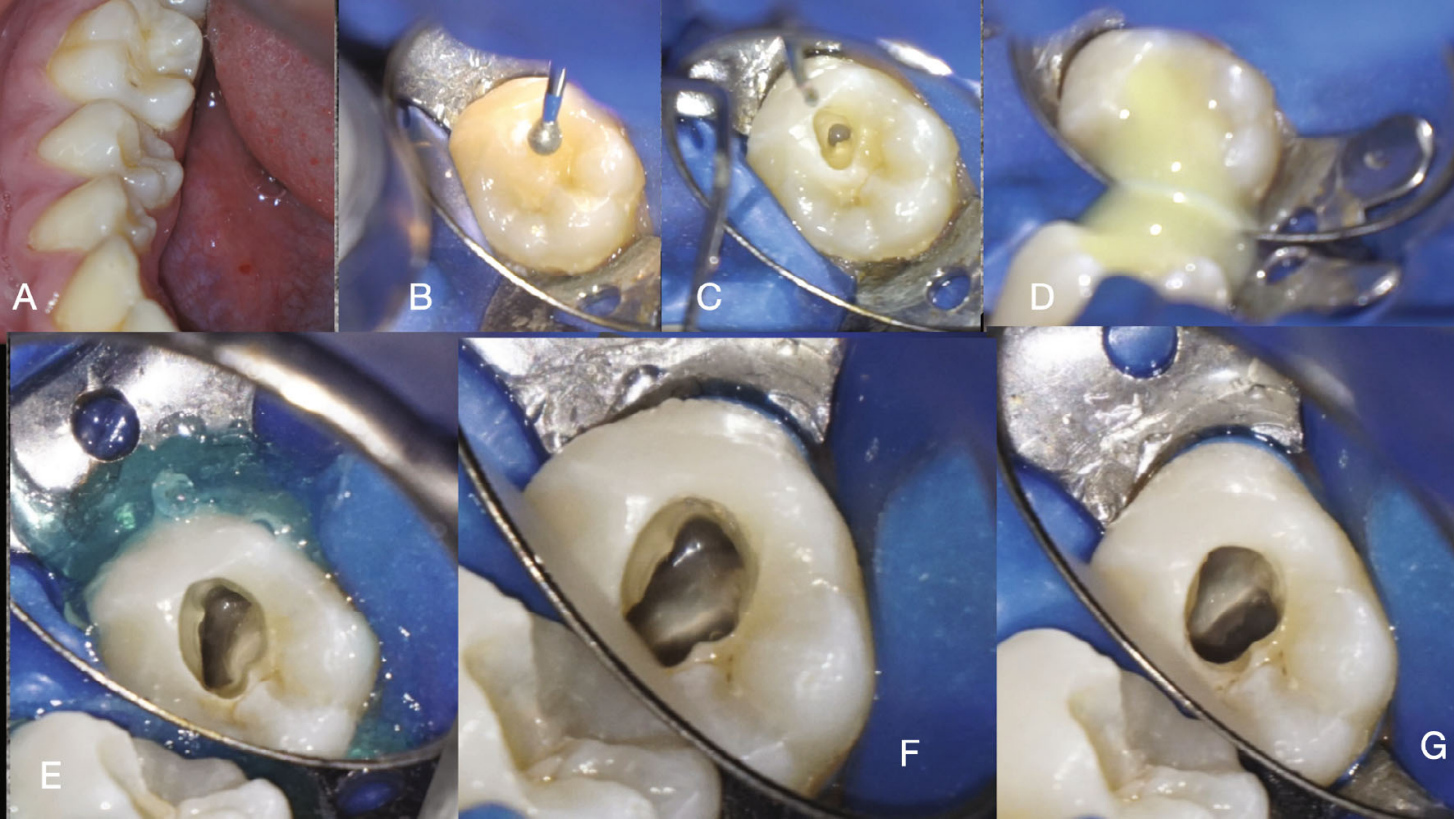
(A) Clinical labial–occlusal view of the second mandibular premolar (buccal cusp attrition). (B) Initial access in the buccal cusp. (C) Initial entry point inside the root canal space that corresponds to the Ax52 CBCT slice. (D) Pus draining from the access cavity. (E) Strategically extended access cavity that corresponds to the Ax49 CBCT slice. (F) Initial access inside the invagination that correspond to the Ax41CBCT slice. (G) Strategically extended intrainvagination access.

The clinical evaluation at the initial visit revealed a buccal fluctuant swelling associated with Tooth 45. The tooth was painful upon percussion, and both thermal and electrical vitality testing were negative. The crown of the second mandibular premolar was larger than its contralateral and included one buccal cusp and three lingual cusps (Figure 2A,B). Radiographic evaluation revealed a Type IIIb DI [6] associated with a periapical lesion (Figure 1A). Because of the complicated nature of the invagination, the previously acquired cone beam examination was retrieved and evaluated (Figure 1B,C) (see Supporting Information 1: Video S1). The sagittal slices revealed the topography and extension of the invagination (Figure 1B). The axial slices revealed the different anatomical compartments and their intercommunications at the different coronoapical levels (Figure 1C). The root canal anatomy projected in the axial slices guided the access cavity preparation for the negotiation of the DI internal anatomy.

**Figure 3:**
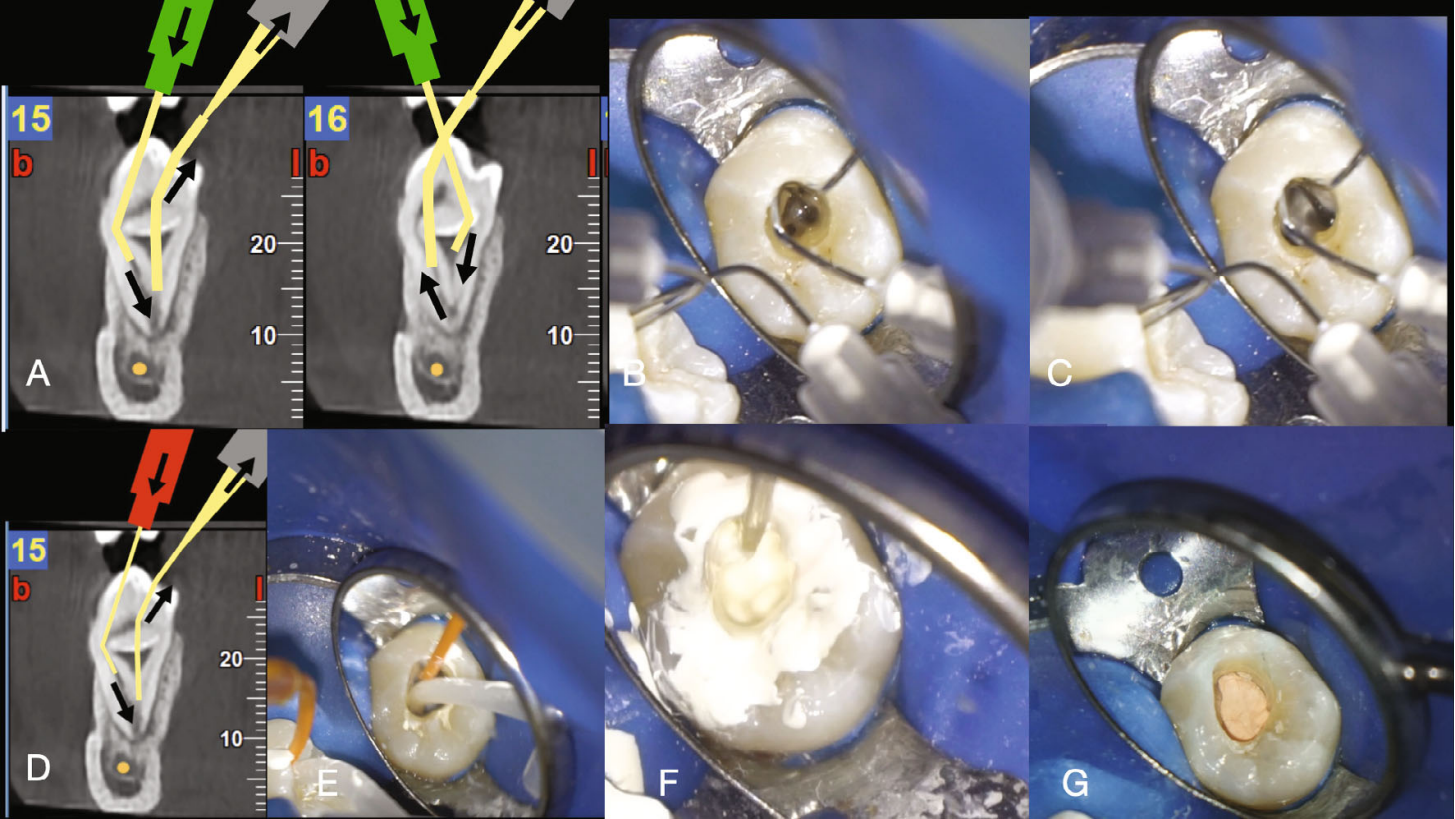
(A) Graphic representation of the injection and evacuation irrigation needle topography on the sagittal CBCT slices (irrigant delivery and evacuation needles shifted from the outer to the inner compartment multiple times). (B) Clinical image of the anatomy filled with irrigant. (C) Clinical image of the irrigant evacuated creating a constant flow of fresh irrigant throughout the anatomy. (D) Graphic representation of the injection and evacuation obturation capillary tips topography on the sagittal CBCT slices (the bioceramic sealer was injected in the outer c‐shaped compartment and evacuated from the central invagination canal). (E) Clinical image of the injection and evacuation obturation capillaries. (F) Clinical image of the dens invaginatus filled with bioceramic sealer and activated with ultrasonics. (G) Clinical image of the injectable gutta‐percha plug.

**Figure 4:**
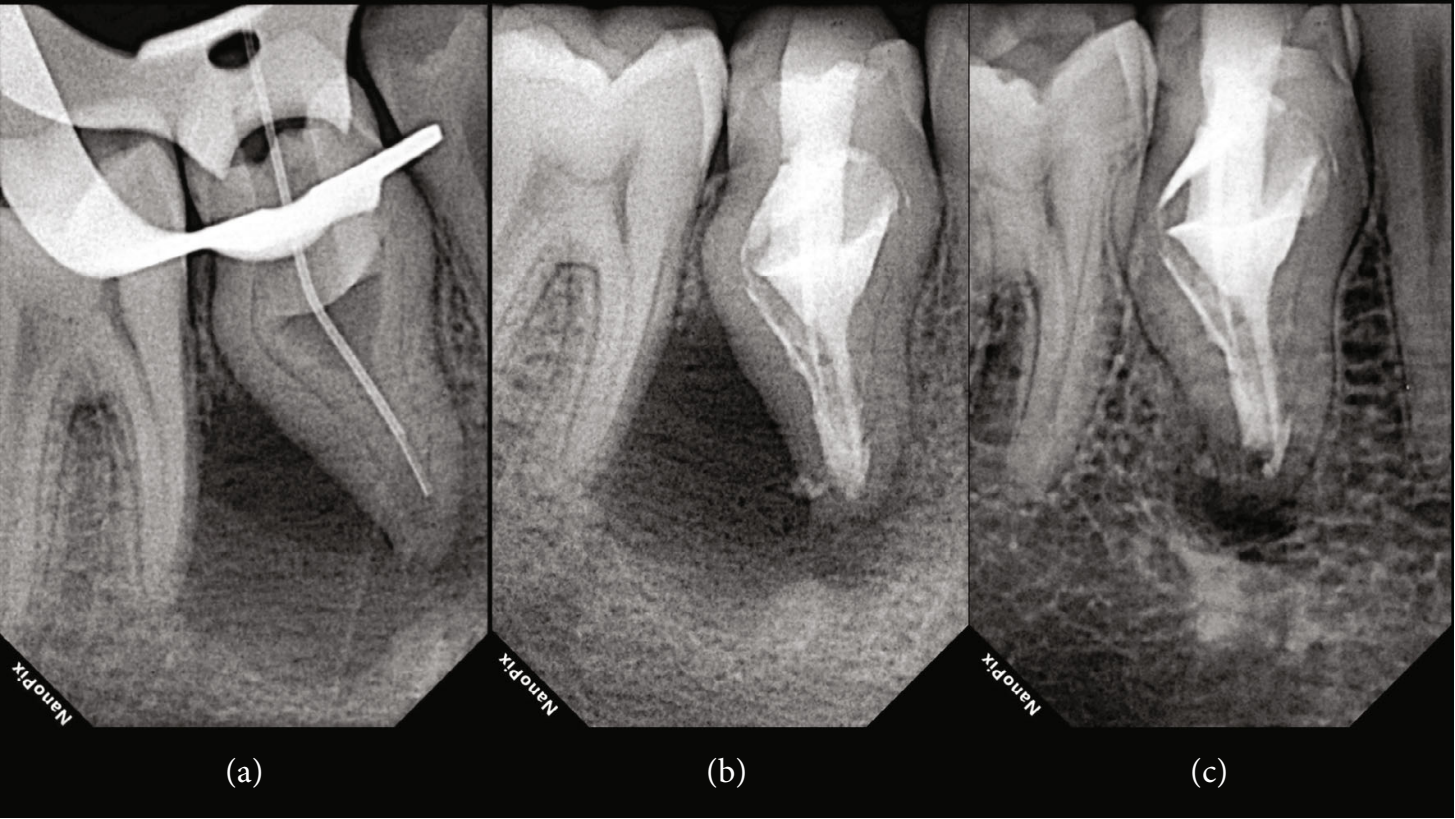
(a) Periapical radiograph with the irrigation needle fitted in the peri‐invagination compartment showing the level of the needle penetration. (b) Postoperative radiograph revealing the anatomy filled with the bioceramic sealer. (c) Four‐year follow‐up radiograph revealing healing of the periapical lesion.

An informed consent was obtained and signed by the parents. The patient was anesthetized with mandibular block anesthesia with 3% mepivacaine (Septodont). The tooth was isolated with a rubber dam (Figure 2B). The initial access cavity preparation was done just below the buccal cusp to correspond with the buccal pulpal space compartment shown in the sagittal and axial cone beam slices (Figures 1B,C, and 2B,C). After the initial entry to the buccal compartment, purulent drainage was noticed (Figure 2D). The tooth was left to drain until the drainage became hemorrhagic and clear. The access cavity was strategically extended to correspond to Axial Slice 49 (Figures 1C and 2E). Through the extended access cavity, the occlusal surface of the invagination was made visible. Under the CBCT imaging guidance, the access cavity was extended apically through the occlusal invagination surface into the intrainvagination space seen in Axial Slice 41 (Figures 1C and 2F,D). As seen in CBCT Axial Slice 49, the pulp canal space had been pushed by the invagination to the periphery of the tooth, creating a semicircular c‐shaped space in the coronal part of the tooth (Figure 1C). In the middle third of the tooth, the c‐shaped space was rendered circular around the invagination and was interrupted by a small peri‐invagination dentinal fusion with the dentinal walls, as seen in Axial Slices 41 and 38 (Figure 1C). In the apical third, the circular pulp canal space was completely engulfing the invagination until both spaces fused in the apical foramen, as seen in Axial Slices 35, 32, 38, and 25 (Figure 1C). The invagination foramen was short of the tooth foramen as seen in Sagittal Slices 15, 16, and 17 (Figure 1B). The internal anatomy of the DI consisted of a central intrainvagination space communicating through the apical third with the circular and semicircular compartments of the pulp canal c‐shaped space. To disinfect the different interconnecting anatomical compartments of the DI, a modified negative‐pressure disinfection procedure was applied (Figure 3A,B,C). A 27‐gauge slotted end needle (Coltene) was fitted through the invagination to reach the apical third. The position of the needle was confirmed with a periapical radiograph (Figure 4a). The needle was attached to the high‐speed suction of the unit through a Luer Lock Adaptor (Ultradent). A second irrigation slotted end 27‐gauge needle was fitted in the external semicircular pulp canal space compartment (Figure 3A). No instrumentation was done. Positive pressure syringe irrigation of a 5% solution of NaOCl was delivered through a 10‐mL syringe in the peri‐invagination compartment, and the intrainvagination suction needle evacuated the irrigant (see Supporting Information 2: Video S2). The positive pressure was arrested each time the compartments flooded until the irrigant could be evacuated from the intrainvagination tube (Figure 3B,C). With this modified negative pressure irrigation (NPI) technique, a continuous flow of irrigant was established through all the internal anatomical compartments of the dens invagination. The delivery of the irrigant was enhanced by shifting the position of the tubes multiple times and changing the flow of the irrigant delivery and evacuation (Figure 3A). Approximately 60 mL of 5% NaOCl was delivered and evacuated through the needles fitted in the different compartments of the DI.

Under negative pressure, the complex anatomical compartments were flooded with 5% sodium hypochlorite solution. UA of the irrigant was achieved by using an irrisafe activation tip (Satelec, Acteon) for 15 s, and the procedure was repeated five times. The power of the ultrasonic was set to 30%, and the ultrasonic tip reached 2 mm short of the estimated length. The total contact time of the NaOCl with the canal walls reached 40 min in total.

The various compartments of the internal anatomy of the DI were dried with capillary suction. Since no instrumentation was performed and thus no smear layer was produced, the use of EDTA irrigation was considered unnecessary.

The root canal system′s obturation was achieved using a modified negative pressure obturation technique coupled with UA of the sealer. A bioceramic delivery capillary tip (Bio‐C sealer, Angelus, Londrina, Brazil) was fitted in the c‐shaped external compartment of the pulp canal space. A second transparent capillary tip was fitted inside the invagination space (Figure 3D,E). The transparent capillary tip was attached in a Luer Lock high‐speed suction adaptor, and the bioceramic delivery tip was fitted to the bioceramic syringe. The suction adaptor was fitted to the high‐speed suction. The bioceramic syringe (Bio‐C sealer, Angelus, Londrina, Brazil) was pressed in a slow, steady way that allowed the continuous delivery of the bioceramic inside the pulp canal semicircular space flowing inside all the compartments until it reached the transparent evacuation suction tip that was fitted in the invagination space (see Supporting Information 2: Video S2). UA of the sealer (irrisafe tip, 10% power) was applied to distribute the sealer better in the lateral extensions of the DI system. Injectable thermoplasticized gutta‐percha was injected over the access cavity, and vertical hydraulic pressure was applied toward the invagination to adapt the bioceramic (Bio‐C sealer, Angelus, Londrina, Brazil) (Figure 3G). The access cavity was restored with composite resin. The whole procedure was done in a single visit since the patient was living on a distant island, and multiple visit appointments were considered extremely difficult to take place.

The postoperative periapical radiograph can be seen in Figure 4b.

**Figure 5:**
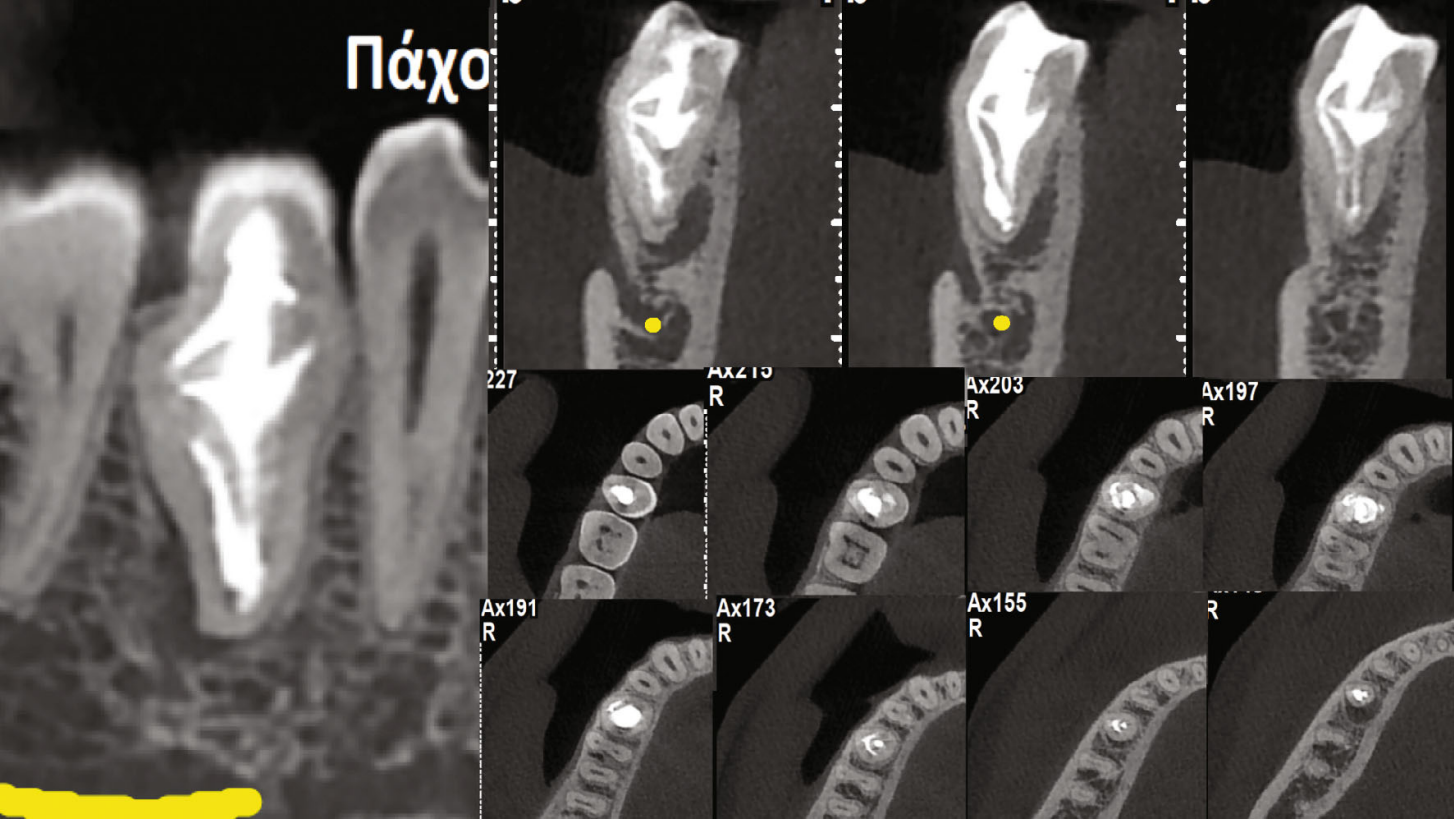
Five‐year follow‐up CBCT radiograph revealing optimal distribution of the obturation material in the interconnecting anatomical compartments of the dens invaginatus Type IIIb and ongoing healing of the periapical/peri‐invagination lesion.

The patient returned for the follow‐up examination 4 years later. The patient was asked to take a follow‐up CBCT scan before the follow‐up appointment; however, he denied because of unnecessary additional radiation concerns. The clinical examination revealed an asymptomatic tooth, and periodontal probing was within normal limits. The radiographic examination revealed healing of the periapical lesion associated with the mandibular premolar DI (Figure 4c). One year later, the patient was contacted again and agreed to have a 5‐year follow‐up CBCT scan (Figure 5). The 5‐year follow‐up clinical examination revealed an asymptomatic tooth, and the periodontal probing was within normal limits. The CBCT evaluation revealed the ongoing healing process (Figure 5). An informed consent for the publication of this case was signed by the patient.

## 3. Discussion

Several management options are available for treating Type IIIb DIs, each with its own considerations and challenges. For cases involving necrotic pulp, both the invagination and the true root canal system should be addressed, with particular emphasis on strategies to manage bacterial infection within the complex anatomy of both.

However, the complex and unpredictable canal morphology of Type IIIb cases makes thorough cleaning and shaping difficult. Traditional instrumentation is obsolete, and irrigation methods may be insufficient to reach all areas of the invagination, leading to incomplete disinfection and potential treatment failure.

To overcome disinfection challenges, a noninstrumentation modified negative pressure disinfection procedure supplemented with UA was selected.

After CBCT mapping of the DI′s anatomical complexity, the interconnecting anatomical compartments were accessed and recorded. For an irrigant solution to be effective, it needs to create a flow to reach the apex and all the anatomical ramifications and carry the particles away [9]. Negative pressure systems allow for the effective delivery of irrigants into complex canal spaces without the risk of extrusion into the periapical tissues, which is critical in Type IIIb cases where the invagination communicates with the periapical area. Additionally, the use of CBCT is essential to fully understand the canal morphology and guide the treatment plan.

In the case reported here, the CBCT‐assessed internal anatomy of the DI is composed of interconnecting compartments. In such anatomies, a modified negative pressure disinfection procedure can be applied. Injecting the disinfectant in one compartment and aspirating from another compartment is an easy and effective way to create a continuous flow of fresh new irrigant solution through the intercommunicating compartments. The capillary tips used for the modified negative pressure disinfection evacuation cycle can be adjusted to offer higher or lower irrigant velocity and shear strength. Negative pressure irrigant delivery systems were found to be effective in safely reaching the working length (WL), without risking extrusion and eliminating the vapor lock effect. UA of the irrigant was found to be able to distribute the irrigant better in the lateral extensions of the root canal system. The combination of both is suggested to provide the best distribution of the irrigants to the complicated anatomy presented here [10, 11].

This combined approach of modified NPI and passive UA might address the limitations of traditional methods, providing superior irrigant delivery and distribution within the complex anatomy of Type IIIb DI [8, 11, 12]. Recently, a prospective cohort study examined the healing outcomes of apical periodontitis after using UA combined with NPI compared to the multisonic ultracleaning system Gentle Wave (GW) (Sonendo, Laguna Hills, CA, United States), which integrates negative pressure, acoustics, and fluid dynamics [13]. The UA technique, when coupled with NPI, was found to be equally effective as the GW multisonic system in promoting apical periodontitis healing [13]. The multisonic ultracleaning system GW (Sonendo, Laguna Hills, CA, United States) is a novel minimally invasive technology that combines negative pressure, acoustics, and fluid dynamics to remove tissue debris and to disinfect the root canal system [13]. GW generates intense hydrodynamic cavitation characterized by a broad spectrum of waves at the high flow rate of 45 mL/min. In vitro studies on GW have demonstrated irrigation safety and a high efficiency of pulp tissue dissolution and biofilm removal even with minimal instrumentation of the root canal. Conventional syringe irrigation (CSI) does not ensure that the irrigation solution reaches the entire WL and the complex anatomy of the canal. Additionally, if taken to the apical third, CSI creates positive pressure, potentially causing the irrigation solution to be forced out of the canal. To enhance the safety and effectiveness of root canal disinfection, alternative contemporary techniques such as NPI, sonic activation, UA, laser‐activated irrigation, and the XPEndo Finisher instruments have been developed. Studies on NPI have reported better penetration of irrigation to WL [10]. In vivo studies have demonstrated effective delivery of irrigation at WL, less debris at 1 mm from WL, and improved reduction of bacterial counts at the apical third of the root canal, compared to CSI. In addition, UA generates acoustic streaming, which enhances the potential of irrigation to contact a greater surface area of the root canal walls. The ultrasonic file oscillation allows for better cleanliness of the root canal system by accessing areas that cannot be touched by instruments, increasing the irrigant temperature and tissue dissolution. The combination of UA and NPI provides the best distribution of the irrigant in the complicated anatomical compartments.

Further research is warranted to assess the long‐term efficacy of this combined approach in disinfecting Type IIIb DI cases.

To seal the internal anatomy, a modified negative‐pressure bioceramic obturation technique was utilized. A flowable bioceramic sealer was applied under negative pressure to fill all the irregular compartments with minimal risk of extrusion beyond the foramen. Additional UA of the sealer was used to distribute and adapt the sealer better into the complex lateral extensions of the root canal system. Sonic activation of the bioceramic sealer resulted in improved penetration compared to no activation [14]. Injectable gutta‐percha can create a customized plugger for complex anatomies, allowing for indirect vertical hydraulic pressure to improve sealer distribution. This marks the first reported application of the modified negative‐pressure bioceramic obturation technique to seal complex anatomical compartments without the risk of extrusion beyond the apex.

Despite the difficulties, root canal therapy can be successful if meticulous disinfection and obturation techniques can address all the challenging anatomical compartments. The 4‐year follow‐up revealed healing of the periapical lesion. The 5‐year CBCT follow‐up radiograph revealed ongoing healing of the periapical lesion and optimal distribution of the obturation material in the highly complex anatomy of the Type IIIb DI.

## 4. Conclusion

The management of Type IIIb DI remains a significant challenge in endodontics due to the complexity of the invaginated anatomy and the associated periapical pathology. Noninstrumented complicated interconnecting anatomical compartments might be effectively sealed after disinfection with the application of the modified negative pressure bioceramic obturation technique. Further research is needed to evaluate this new technique and investigate its possible applications.

This case report has been written according to the Preferred Reporting Items for Case reports in Endodontics (PRICE) 2020 guidelines [15].

## Conflicts of Interest

The authors declare no conflicts of interest.

## Funding

No funding was received for this manuscript.

## Supporting Information

Additional supporting information can be found online in the Supporting Information section.

## Supporting information


**Supporting Information 1** Video S1: CBCT mapping of the anatomical compartments of the dens in dente Type IIIb.

**Supporting Information 2** Video S2: Positioning of the injection and evacuation needles for negative pressure disinfection and bioceramic obturation.

## Data Availability

The data that support the findings of this study are available from the corresponding author upon reasonable request.
